# Bacterial Nanocellulose Hydrogels as a Next-Generation Biomaterial for Cardiac and Vascular Tissue Engineering: Structural, Biological, and Translational Perspectives

**DOI:** 10.3390/gels12060474

**Published:** 2026-05-29

**Authors:** Otávio Simões Girotto, Maria Angelica Miglino, Giovanna Ayumi M. Fukuda, Caliandra Bernardi, Cristiane Lurdes Paloschi, Talissa Caroline Pollon, Fernando Gonçalves da Silva Petronio, Fernando Chissico, Matheus Henrique Herminio Garcia, Vinicius Gabriel Silverio Scholl, Sandra Maria Barbalho, Rogerio Leone Buchaim, Daniela Vieira Buchaim, Vivien Patricia Garbin, Samara Silva de Souza

**Affiliations:** 1 Regenerative Medicine Laboratory “Carlos Augusto Camargo de Souza Baptista”, Universidade de Marília (UNIMAR), Marília 17525-902, SP, Brazil; otgirotto@gmail.com (O.S.G.); talissapollon@gmail.com (T.C.P.); fernandogspetronio@gmail.com (F.G.d.S.P.); cfernandochissico@gmail.com (F.C.J.); matheushenrigarcia@gmail.com (M.H.H.G.); vinicius.scholl@hotmail.com (V.G.S.S.); 2 Postgraduate Program in Biotechnology, Universidade Tecnológica Federal do Paraná, Dois Vizinhos 85660-000, PR, Brazil; giovannayumi21@gmail.com (G.A.M.F.); bernardi.caliandra@gmail.com (C.B.); cpaloschi@utfpr.edu.br (C.L.P.); samarasouza@utfpr.edu.br (S.S.d.S.); 3 School of Medicine, Universidade de Marília (UNIMAR), Marília 17525-902, SP, Brazil; smbarbalho@gmail.com; 4 Department of Biological Sciences, Bauru School of Dentistry (FOB/USP), University of Sao Paulo, Bauru 17012-901, SP, Brazil; rogerio@fob.usp.br; 5 Medical School, University Center of Adamantina (FAI), Adamantina 17800-000, SP, Brazil; danibuchaim@alumni.usp.br; 6 Department of Postgraduate, Dentistry School, Faculty of the Midwest Paulista (FACOP), Piratininga 17490-010, SP, Brazil; 7 Postgraduate Program in Clinical Research, São Paulo State University (UNESP-Universidade Estadual Paulista), Botucatu 18610-307, SP, Brazil; 8 Laboratory of Basic Stem Cell Biology (LABCET), Instituto Carlos Chagas/Fiocruz-PR, Curitiba 80210-170, PR, Brazil; viviengarbin@gmail.com

**Keywords:** bacterial nanocellulose, hydrogels, cardiac tissue engineering, vascular grafts, bioinks, 3D bioprinting, mechanotransduction

## Abstract

Since current therapies cannot regenerate lost myocardium or reverse adverse ventricular remodeling—major contributors to worldwide cardiovascular mortality—advanced biomaterials, particularly hydrogels, have emerged as promising therapeutic platforms. Among these, bacterial nanocellulose (BNC) has gained increasing attention due to its hydrated nanofibrillar architecture, high crystallinity, robust mechanical performance, and excellent water-retention capacity, features that closely resemble key aspects of the native extracellular matrix. These properties provide a favorable microenvironment for cell adhesion, survival, and tissue organization in cardiovascular applications. Preclinical evidence suggests that BNC-based cardiac constructs, including acellular patches and cell-laden systems, may reduce post-infarction ventricular dilation, promote angiogenesis, and improve cellular engraftment. In vascular tissue engineering, BNC has also been explored in small-diameter grafts, anisotropic hydrogel systems, and shape-memory conduits with encouraging hemocompatibility and functional durability. Functional modifications—including gelatin incorporation, oxidative surface treatments, peptide grafting, conductive polymers, and structural alignment strategies—further expand the biological and mechanical versatility of BNC-based systems. In addition, BNC-containing bioinks have demonstrated promising rheological behavior, printability, and cell compatibility for 3D bioprinting applications. Despite these advances, important challenges remain, including optimization of material functionalization, host integration, degradation control, vascularization, scalable manufacturing, and regulatory translation toward clinical application.

## 1. Introduction

Cardiovascular diseases remain the leading cause of mortality worldwide [1], driven by myocardial infarction (MI), chronic ischemia, and progressive heart failure. Following an ischemic event, the adult mammalian myocardium exhibits limited regenerative capacity, resulting in cardiomyocyte death, thinning of the ventricular wall, adverse remodeling, fibrosis, and chamber dilatation [2]. Conventional therapies—including pharmacological management, stenting, and surgical revascularization—can attenuate acute injury but do not replace lost myocardium, highlighting the need for biomaterials capable of supporting cardiac repair [3,4].

A broad range of nanomaterials and biomaterials, including polymers, metal oxides, graphene-based systems and hybrid composites, has been investigated for cardiovascular tissue engineering [5]. Biomaterials constitute a promising alternative to organ transplantation, circumventing limitations such as donor shortage and immune rejection [6], and their demand has increased significantly in recent years [1]. Among natural biomaterials, polysaccharide- and protein-based systems have gained particular relevance due to their biocompatibility, biodegradability and sustainability, with hydrogels standing out—three-dimensional polymeric networks capable of absorbing and retaining water in their three-dimensional structure—supporting applications in vascular grafts, controlled drug release, and myocardial reconstruction [1,5].

Bacterial nanocellulose (BNC), produced mainly by the bacterium *Komagataeibacter xylinus*, is a polysaccharide characterized by high tensile strength, elevated water content, a highly crystalline structure and an ultrafine, highly pure nanofibrillar network [7,8,9]. These features distinguish BNC among bacterial biopolymers and have prompted extensive investigation of its biomedical applications [10]. Beyond its structural advantages, BNC offers practical benefits: it can be produced at scale with low cost, and the fermentation process can utilize agricultural and animal-derived residues as substrates, reducing environmental impact and improving sustainability [11,12]. Recent literature also highlights that nanocellulose, in its various forms, has emerged as a promising next-generation biomaterial due to its biocompatibility, chemical versatility, and ability to be tailored for complex biomedical applications [13,14].

Multiple studies have demonstrated that BNC exhibits excellent compatibility with a range of human cell types, including fibroblasts, keratinocytes, osteoblasts, endothelial cells and mesenchymal stem cells, and that this compatibility can be further enhanced by physical or chemical modifications designed to improve cell adhesion and viability [13]. These characteristics underlie its successful use across biomedical fields such as skin regeneration, vascular grafting and in vitro barrier models. Notably, BNC-based membranes have shown superior performance in blood–brain barrier models, including enhanced transepithelial electrical resistance compared with conventional membranes [15].

**Figure 1:**
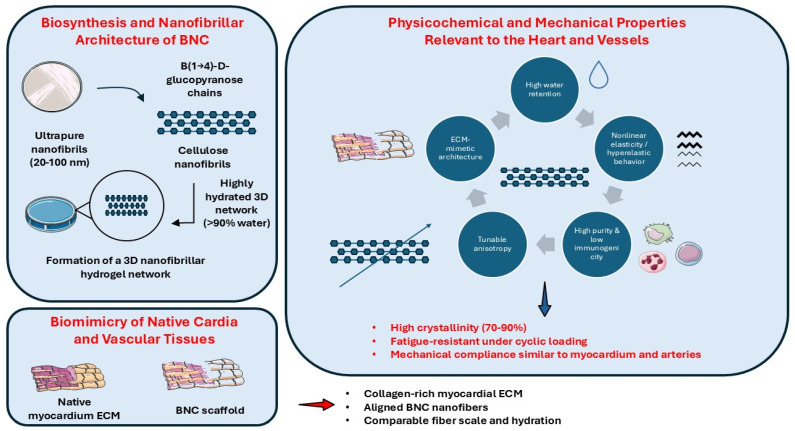
Biosynthesis, nanofibrillar architecture, and cardiovascular relevance of bacterial nanocellulose (BNC). BNC is biosynthesized as β-(1→4)-D-glucopyranose chains that assemble into ultrapurified nanofibrils (20–100 nm), forming a highly hydrated three-dimensional network (>90% water). This nanostructure closely mimics the extracellular matrix (ECM) of native cardiac and vascular tissues. Key physicochemical and mechanical properties—including high crystallinity, water retention, nonlinear elasticity, tunable anisotropy, and low immunogenicity—support its use in cardiovascular applications. These features enable mechanical compliance similar to myocardium and arteries, promote cell compatibility, and facilitate biomimetic scaffold design for cardiac and vascular tissue engineering.

Recent findings highlight that BNC displays substantial potential for the fabrication of artificial blood vessels, especially in ready-to-use vascular graft formats [7,13]. Its physicochemical and mechanical characteristics facilitate integration with biological tissues and maintain performance under physiological flow and pressure. Mechanical suitability—namely high tensile strength, elasticity and creep resistance—is essential for cardiovascular implants, including vascular grafts and heart valve structures, and BNC meets many of these requirements [13]. An overview of BNC biosynthesis, structural organization, and key physicochemical properties relevant to cardiac and vascular tissue engineering is presented in Figure 1.

In cardiac regeneration, BNC-based constructs have gained increasing attention. Membrane patches loaded with cocultured cells have demonstrated cardioprotective effects in post-infarction models, preserving cell viability and attenuating adverse remodeling [16]. Likewise, BNC has shown promise as a cardiac patch capable of reducing ventricular dilation, supporting therapeutic cell engraftment and stimulating angiogenesis. Its utility extends to vascular tissue engineering as well, where BNC has been evaluated as a small-caliber vessel substitute, as a component of anisotropic composite hydrogels, and as a shape-memory conduit showing hemocompatibility, endothelialization and long-term patency in vivo [17,18,19].

Collectively, these properties position BNC as a next-generation biomaterial for cardiac and vascular engineering. Although BNC can be processed into multiple structural formats, including membranes, patches, conduits, composite scaffolds, and printable formulations, these architectures are all derived from the same highly hydrated nanofibrillar hydrogel network. In this review, the term BNC hydrogel is therefore used in a broad biomaterials sense to describe BNC-based systems whose biological and mechanical performance depends on water-rich fibrillar architecture, even when the final construct is presented as a membrane, patch, or vascular conduit. The following sections review the physicochemical, mechanical, and biological features of BNC, together with its applications in cardiac patches, myocardial scaffolds, vascular grafts, and bioinks for bioprinting, highlighting its unique advantages for cardiovascular tissue regeneration.

## 2. Properties of Bacterial Nanocellulose (BNC) Relevant to Cardiac Engineering

### 2.1. Structure and Composition

BNC is a linear β-(1→4)-D-glucopyranose polymer biosynthesized by *Komagataeibacter* spp., forming an ultrapurified nanofibrillar network free of lignin, hemicelluloses, and extractives typically found in plant cellulose [8,11,19,20,21]. This purity is a central advantage for biomedical use, as it minimizes immunogenicity and eliminates the need for harsh chemical treatments [1,17]. The nanofibrils of BNC typically range from 20 to 100 nm in diameter, forming a highly entangled, three-dimensional hydrogel-like architecture that closely resembles collagen fibrils of the native myocardial extracellular matrix [8,20,22,23].

BNC forms smooth, highly interconnected nanofibrillar networks with hydration levels frequently exceeding 90–99%, a characteristic that enables efficient nutrient diffusion and gas transport through the scaffold [24]. Its nanostructure exhibits high crystallinity, typically between 70% and 90%, resulting from dense intermolecular hydrogen bonding within its fibrillar architecture [22,25,26]. These structural features contribute to the material’s water-retention capacity, which creates a hydrated microenvironment highly compatible with cardiac, endothelial, and stem cell populations [22]. Additionally, BNC presents intrinsic potential for nanofibril alignment, which can be controlled during biosynthesis or through hydrogel formulation; this alignment capability is particularly valuable for cardiac engineering, as it allows the generation of scaffolds whose anisotropy more closely mimics that of native myocardial tissue. Furthermore, recent reviews emphasize that BNC possesses significant potential for chemical and structural modification, enabling fine-tuning of its physicochemical properties for specific biomedical applications [22,27].

### 2.2. Mechanical Properties

The mechanical performance of BNC is one of the principal reasons for its growing relevance in cardiac tissue engineering. Owing to its densely interconnected nanofibrillar network and high crystallinity, BNC exhibits a unique combination of stiffness, elasticity, and fatigue resistance that allows it to withstand physiological deformation while maintaining structural integrity [28]. Pure BNC presents nonlinear elastic behavior characteristic of soft biological tissues, a property attributed to its hydrated nanofiber architecture and extensive hydrogen bonding among fibrils [29]. Modeling studies have demonstrated that BNC conforms to hyperelastic constitutive laws, indicating its suitability for predicting strain distribution and wall mechanics when applied to the beating myocardium [30,31].

In native hydrated form, BNC retains its toughness and flexibility even under cyclic loading, enabling its use as a reinforcing patch in infarcted myocardium [32,33]. This capacity is confirmed by in vivo evidence showing that BNC patches limit post-infarction ventricular dilation and help preserve left ventricular geometry, suggesting that the material mechanically stabilizes the injured wall during the remodeling process [16]. Such stabilization is critical in the early stages following myocardial infarction, when thinning of the ventricular wall and excessive strain typically lead to progressive functional decline [31,34].

Quantitative comparison across representative BNC-based cardiovascular systems further highlights the material’s tunability. Gelatin-mixed BNC scaffolds developed for cardiomyocyte culture exhibit elastic moduli of approximately 75 kPa, closely approximating myocardial mechanical compliance and supporting cardiomyocyte spreading and maturation [34]. In contrast, native BNC generally exhibits substantially higher stiffness due to its highly crystalline nanofibrillar architecture, favoring applications requiring structural reinforcement, such as infarct stabilization patches [28,29]. PVA–BNC composites designed for vascular applications display anisotropic compliance and nonlinear strain responses approaching native arterial mechanics, making them more suitable for vascular graft engineering [27]. Shape-memory and multilayer vascular conduits further demonstrate the capacity of BNC-derived systems to tolerate physiological pulsatile loading while maintaining luminal patency [18,35]. These quantitative differences emphasize both the broad mechanical tunability of BNC-based systems and the persistent lack of standardized benchmarking across cardiovascular biomaterials.

When combined with polyvinyl alcohol (PVA), BNC hydrogels acquire anisotropic mechanical behavior that closely parallels the compliance and strain response of the native aortic wall, making the composite attractive for both vascular and myocardial constructs [27]. Integration with gelatin reduces stiffness and enhances viscoelasticity while simultaneously improving surface roughness, thereby creating a microenvironment more permissive to cardiomyocyte adhesion and spreading [34]. Additional modifications, such as blending with starch (BNC/PS), yield conduits with dual-layer structures capable of sustaining physiological pressures and maintaining patency in vivo, demonstrating their suitability for small-caliber vascular grafts [35].

To provide a clearer comparative overview, representative BNC-based systems and their key physicochemical, mechanical, and biological features are summarized in Table 1.

Overall, this comparison illustrates that BNC-based systems are not mechanically uniform; rather, their performance depends strongly on formulation, functionalization, and intended cardiovascular application.

Advanced architectures, including multilayered or shape-memory BNC constructs, have also been engineered to accommodate dynamic deformation and recover form after mechanical stress, supporting their use in both cardiac and vascular interventions [18]. Collectively, these findings reveal that BNC possesses an intrinsically biomimetic mechanical profile that can be further tailored to match specific cardiac or vascular tissue requirements. Its ability to combine strength, elasticity, and durability under physiological conditions supports its potential as an adaptable platform for myocardial reinforcement, engineered cardiac tissues, and implantable cardiovascular devices.

### 2.3. Biocompatibility and Cell–Material Interactions

BNC has demonstrated excellent biocompatibility across in vitro and in vivo models, primarily due to its ultrapure composition, high water content, and nanofibrillar morphology. Unlike plant-derived cellulose, BNC is free of lignin, hemicellulose, and other extractives, thereby minimizing the risk of immunogenic or inflammatory responses and enabling more predictable cellular behavior upon implantation [25]. Its three-dimensional nanofibrillar architecture forms an interconnected porous hydrogel that closely mimics the native extracellular matrix (ECM), facilitating efficient nutrient diffusion while providing structural support for a broad range of cardiac-relevant cell types [31].

In vitro studies repeatedly confirm that BNC supports robust cell adhesion, growth, and viability. Muscle-derived cells and mesenchymal stromal cells maintain viability levels exceeding 90% during two-week culture periods on BNC membranes, with scanning electron microscopy demonstrating extensive spreading and formation of cell–fiber anchoring structures that extend across the BNC network [16]. These findings are further corroborated by endothelial and fibroblast studies, where BNC scaffolds promote cytoskeletal organization and encourage phenotypes resembling native tissue, including enhanced endothelial differentiation profiles [36]. Together, these data indicate that BNC not only supports cellular survival but may actively influence differentiation trajectories relevant to cardiovascular regeneration.

Cardiomyocyte adhesion to unmodified BNC is more limited, reflecting the absence of intrinsic integrin-binding motifs such as RGD. However, the material’s surface chemistry, dominated by hydroxyl groups, readily adsorbs proteins from serum or ECM supplements, forming a bioactive conditioning layer that partially compensates for the lack of peptide ligands [29]. This mechanism underlies the moderate adhesion observed on pure BNC and becomes significantly enhanced when the material is combined with biomolecules. Biochemical functionalization, particularly gelatin incorporation, further enhances cardiomyocyte adhesion by introducing integrin-binding motifs and improving nanoscale bioactivity, as discussed in later sections [34]. Surface modification through oxidative functionalization or conjugation with positively charged residues, such as arginine, also improves protein adsorption and cell anchoring by introducing carboxylate groups that strengthen electrostatic interactions [37].

The interaction between cardiac cells and their mechanical microenvironment is critical for guiding sarcomeric organization and contractile behavior, with studies showing that variations in stiffness and topography strongly influence cardiomyocyte maturation [38].

Conductive BNC composites offer further enhancements relevant to electrically active cardiac cells. Incorporation of polypyrrole (PPy) into BNC scaffolds yields hybrid matrices that support well-spread cardiomyoblasts with extended filopodia and promote features associated with electrical coupling and maturation [39]. These conductive composites suggest a promising avenue for engineering bioelectrically integrated myocardial tissues or patches capable of interacting with the heart’s conduction system.

Biocompatibility is equally strong in vivo. BNC membranes implanted on infarcted myocardium integrate without eliciting significant inflammation, fibrosis, or foreign-body reactions. In myocardial infarction models, acellular BNC patches reduce ventricular dilation, partially preserve ventricular geometry, and promote angiogenesis within the infarct zone, likely through mechanobiological stabilization and recruitment of endogenous reparative cells [16]. When loaded with therapeutic cells, BNC enhances engraftment and supports the retention of proliferative and angiogenic populations, as indicated by BrdU and VEGF expression analyses. Vascular studies also demonstrate excellent hemocompatibility: BNC-based conduits exhibit patency, minimal thrombogenicity, and progressive endothelialization in small-caliber vascular implants, even forming multilayered neovessel-like structures in medium-term studies [18,40].

Taken together, these observations establish BNC as a uniquely biocompatible cardiovascular scaffold that interacts favorably with cardiac, endothelial, and stromal cells. Its nanofibrillar topography, hydrophilic chemistry, and capacity for biochemical modification provide a versatile platform for promoting cell adhesion, guiding phenotype, and supporting tissue remodeling. The combination of these properties positions BNC as an exceptionally promising material for engineered myocardial constructs, regenerative cardiac patches, vascular grafts, and hybrid bioelectronic systems.

## 3. BNC as Scaffolds for Cardiomyocytes and Cardiac Tissue Regeneration

BNC has emerged as a versatile platform for cardiac tissue engineering because it can be used both as an in vivo reinforcing patch for infarcted myocardium and as an in vitro scaffold for cardiomyocyte culture and maturation. Importantly, BNC membranes and patches should not be interpreted as dry films, but rather as hydrated nanofibrillar hydrogel-like constructs whose high water content, porosity, and fibrillar organization govern their interaction with cardiac cells and injured myocardium. Its ECM-mimetic fibrillar structure, tunable mechanics, and modifiable surface chemistry allow BNC to be adapted for different stages of the regenerative process: mechanical stabilization of the ventricular wall, delivery and retention of therapeutic cells, support of endogenous tissue remodeling, and construction of pre-formed cardiac tissues.

### 3.1. In Vitro Support of Cardiomyocytes and Cardiomyoblasts

Initial investigations using neonatal rat ventricular cardiomyocytes (nr-vCMCs) indicated that pristine BNC membranes alone are not optimal substrates for these highly specialized cells: cardiomyocytes attached, but with limited spreading and reduced viability compared with more biomimetic formulations [34]. This limitation is consistent with the absence of intrinsic cell-adhesion peptide motifs in native cellulose, despite the presence of abundant hydroxyl groups that can adsorb serum proteins.

To overcome this, BNC has been combined with biomacromolecules that introduce biochemical cues while modulating mechanical and topographical properties. Salehghamari et al. produced three scaffold types: pure BNC, gelatin-coated BNC (BCG) and gelatin-mixed BNC (mBCG). Neonatal cardiomyocytes cultured on these scaffolds showed the highest viability, spreading and maintenance of spindle-shaped morphology on mBCG, followed by BCG, while pure BNC supported the lowest cell density. Atomic force microscopy analysis reported that mBCG exhibited intermediate nanoscale roughness, which correlated with improved cardiomyocyte adhesion; cardiomyocytes are known to prefer moderately rough surfaces that enhance protein adsorption and integrin-mediated attachment [41]. Mechanically, mBCG presented tensile strength (~11.6 kPa), elongation (~12.6%) and elastic modulus (~75 kPa) values closer to native myocardial ranges than the stiffer BNC and BCG scaffolds, indicating that both mechanical compliance and surface nanoarchitecture cooperate to support cardiomyocyte function.

Electroconductive BNC composites further illustrate how the material can be tailored for cardiac cells. In a study using cardiomyoblast H9c2 cells, plain BNC substrates led to low cell density, limited filopodia formation and abnormal rounded morphology, whereas BNC–polypyrrole (BNC–PPy) scaffolds promoted extensive cell spreading, abundant filopodia and cellular alignment along the nanofibers. Cardiomyoblasts on BNC–PPy retained a more characteristic cardiac-like phenotype and showed improved expression of cardiac markers under differentiation conditions, suggesting that the combination of BNC’s structural support with PPy’s electrical conductivity can enhance cytoskeletal organization and potentially favor electromechanical maturation. These results align with broader evidence that conductive biomaterials can promote myocardial tissue maturation, sarcomeric organization and synchronization of spontaneous beating [33].

Beyond direct cardiomyocyte culture, BNC has also been explored as a substrate in hybrid cellulose-based hydrogels that recapitulate myocardial ECM composition. Cellulose hydrogels, including those derived from BNC, have been combined with decellularized myocardial matrix and other bioactive components to produce injectable or pre-formed scaffolds that support differentiation of stem cells into cardiomyocyte-like phenotypes and promote integrin-mediated signaling important for cardiac lineage specification [42,43]. In this context, BNC contributes mechanical robustness and nanoarchitecture, while ECM components provide biochemical specificity.

### 3.2. BNC-Based Patches for Myocardial Repair In Vivo

The application of BNC patches directly onto the infarcted myocardium represents one of the most advanced uses of this biomaterial in cardiac regeneration. In a preclinical rat model of myocardial infarction, BNC membranes were cultured for 14 days with a coculture of skeletal muscle cells and bone marrow–derived mesenchymal stem cells, then implanted over the infarcted region, either with or without the cellular component [16]. Histological analysis confirmed that cocultured cells adhered to the BNC surface, adopted a skeletal-like morphology and proliferated during the culture period, while scanning electron microscopy demonstrated dense coverage of the BNC membrane by cells before implantation.

Echocardiographic follow-up revealed that BNC patches, even in the acellular group, preserved left ventricular dimensions significantly better than untreated infarcted controls, indicating a clear mechanical stabilization of the ventricular wall. Although left ventricular ejection fraction did not improve in the acellular group, the prevention of chamber dilatation and wall thinning reflects attenuation of adverse remodeling. When BNC was combined with therapeutic cells, additional benefits were observed at the histological level, including increased BrdU-positive proliferative cells and enhanced angiogenesis, as shown by VEGF immunostaining within the infarct scar and at the BNC–tissue interface. These results suggest that BNC simultaneously provides mechanical support to the injured wall and acts as a delivery platform that retains paracrine-active cells at the site of injury [16].

The favorable host response to BNC in this setting is supported by minimal inflammatory infiltration, absence of significant fibrotic encapsulation and progressive integration at the interface between the patch and native myocardium. These characteristics are consistent with observations from other implantation sites, where BNC demonstrated low immunogenicity and stable tissue integration, and underscore its promise as a mechanically competent, biologically quiet cardiac patch.

### 3.3. Mechanisms of Cardiomyocyte Interaction with BNC

The interaction of cardiomyocytes with BNC is governed by a combination of nanotopographical cues, surface chemistry and, when present, conductive or bioactive modifications. The intrinsic three-dimensional nanofibrillar structure of BNC promotes cell anchoring and helps maintain a more differentiated phenotype compared to smooth, non-nanostructured surfaces, which tend to induce cell dedifferentiation [44]. The high surface area of the nanofibers facilitates adsorption of adhesion proteins such as fibronectin and vitronectin from serum or conditioned media; these proteins then engage integrins on cardiomyocytes and stromal cells, enabling focal adhesion formation and downstream signaling.

Chemically, the dense population of hydroxyl groups on BNC renders the surface highly hydrophilic and capable of forming hydrogen bonds with adsorbed proteins and ECM molecules. These interactions are further enhanced when BNC is oxidized to introduce carboxylate (–COO^−^) groups, which provide additional negatively charged sites for electrostatic binding of positively charged protein domains and growth factors [37]. When combined with gelatin, BNC scaffolds gain RGD-containing motifs that directly engage integrins; this modification explains the superior adhesion, viability and spreading of neonatal cardiomyocytes on gelatin-mixed BNC compared with unmodified BNC or merely coated surfaces [34]. Beyond initial adhesion, these cell–material interactions activate mechanotransduction pathways central to cardiac maturation. Integrin engagement promotes focal adhesion kinase (FAK) signaling, cytoskeletal tension, and downstream mechanosensitive pathways such as YAP/TAZ, which regulate cardiomyocyte spreading, alignment, and phenotypic maturation. Because cardiomyocytes are highly sensitive to matrix stiffness, substrates that more closely approximate myocardial mechanical compliance can better support sarcomeric organization and contractile behavior. Cytoskeletal remodeling induced by nanoscale topography also influences intracellular calcium handling, which is essential for excitation–contraction coupling and synchronized cardiac function.

Hybridization with decellularized extracellular matrix (dECM) may further enhance these mechanisms by supplying tissue-specific biochemical cues absent in native BNC. Because cardiac dECM retains structural proteins and adhesion ligands characteristic of the native myocardial microenvironment, combining BNC with dECM represents a promising strategy to unite structural robustness with tissue-specific biochemical functionality for cardiomyocyte support.

**Figure 2:**
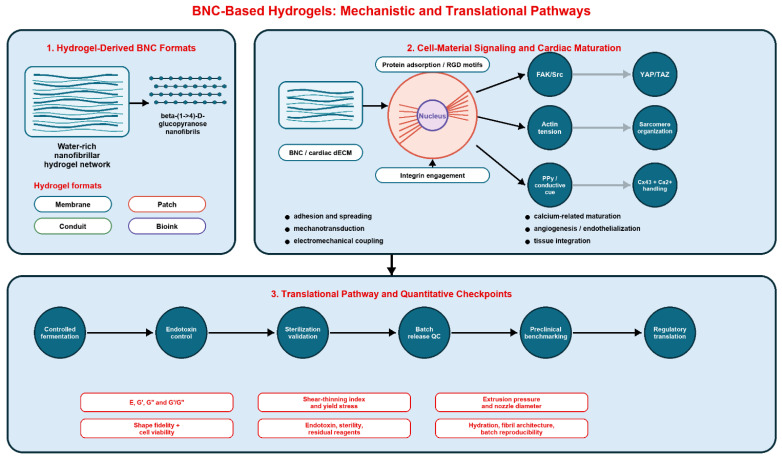
Mechanistic and translational pathways of bacterial nanocellulose (BNC)-based hydrogels in cardiovascular regeneration. BNC-based membranes, patches, conduits, and bioinks are interpreted as hydrated nanofibrillar hydrogel architectures rather than dry films. At the cell–material interface, BNC and hybrid BNC–cardiac decellularized extracellular matrix (dECM) systems support protein adsorption, RGD-mediated or ECM-mediated integrin engagement, cytoskeletal organization, and mechanotransduction through pathways such as FAK/Src and YAP/TAZ signaling. Conductive modifications, including PPy-containing systems, may further contribute to electromechanical coupling, calcium-related maturation, and sarcomere organization. Translational development requires standardized quantitative checkpoints, including E, G′, G″, G′/G″ ratio, shear-thinning behavior, extrusion pressure, nozzle diameter, shape fidelity, post-printing viability, endotoxin control, sterility, residual reagent assessment, hydration, fibril architecture, and batch-to-batch reproducibility. BNC, bacterial nanocellulose; dECM, decellularized extracellular matrix; FAK, focal adhesion kinase; PPy, polypyrrole.

Electrical and mechanical cues also play important roles. Conductive BNC–PPy composites enable more physiological electrical communication between cultured cells and their substrate, which has been associated with improved sarcomeric organization and expression of cardiac markers in H9c2 cells relative to non-conductive BNC [45]. Similarly, aligning BNC nanofibers or creating anisotropic composite hydrogels allows cardiomyocytes to orient along preferred directions, supporting the unidirectional growth and contraction patterns needed for functional myocardium [46]. Thus, the combination of biochemical ligands, nanoscale architecture, surface charge and mechanical anisotropy defines a multidimensional design space through which BNC can be optimized for cardiomyocyte adhesion, maturation and integration (Figure 2).

### 3.4. Implications for Clinical Translation

Taken together, current evidence positions BNC as a highly promising material for myocardial repair strategies. As an acellular patch, BNC already suggests clinically relevant benefits by mechanically reinforcing the infarcted wall and mitigating adverse ventricular remodeling [16]. When combined with therapeutic cells, bioactive proteins or angiogenic factors, BNC additionally serves as an efficient delivery and retention platform, potentially enhancing paracrine signaling and local regeneration. Gelatin-modified and conductive BNC scaffolds further extend their utility toward the fabrication of engineered cardiac tissues, where control over cell adhesion, phenotype and electrical coupling is paramount [34]. However, current evidence remains predominantly preclinical and heterogeneous, with substantial variability in scaffold formulation, implantation models, outcome metrics, and follow-up duration, which limits direct comparison across studies and delays robust translational benchmarking. A concise overview of key BNC-based systems for cardiac applications is provided in Table 2.

Overall, BNC occupies a unique position at the intersection of multifunctional cardiovascular biomaterial platform. Its capacity to be processed as membranes, hydrogels, conduits, and composite scaffolds, together with its proven performance in preclinical myocardial infarction models, supports its candidacy for future clinical translation as a cardiac patch and as a building block for engineered myocardial tissues and combined cardiac–vascular implants [47,48].

These findings align with broader cardiac tissue engineering strategies that integrate structural biomaterials, biochemical cues, and electromechanical considerations to restore functional myocardium after injury [49].

## 4. BNC for Vascular Tissue Engineering and Cardiovascular Integration

BNC suggests strong potential for vascular tissue engineering and for constructing hybrid cardiac–vascular systems. This is particularly relevant because myocardial regeneration requires not only mechanical stabilization of the infarcted wall but also restoration of microvascular perfusion. The same features that make BNC suitable for myocardial patches, biocompatibility, porous nanofibrillar architecture, mechanical resilience and chemical modifiability, directly support its function as a vascular scaffold.

Similarly, BNC-based vascular grafts and conduits retain the defining features of hydrogel biomaterials, including high hydration, permeability, compliant deformation, and a water-rich fibrillar interface with blood and vascular cells.

### 4.1. Structural Suitability of BNC for Vascular Grafts

Experimental vascular conduits produced from BNC display structural characteristics that mimic small-caliber arteries. Liu et al. [50] demonstrated that hierarchical BNC/potato starch vascular grafts possess a bilayer architecture comprising a smooth luminal surface and a porous outer layer, supporting physiological blood flow with approximately 75% in vivo patency and resistance to aneurysmal deformation.

Additional engineering strategies include multilayer and shape-memory BNC conduits. These constructs are capable of recovering their original shape after deformation and can withstand pulsatile loading without structural failure, reinforcing their potential application as coronary or peripheral bypass grafts. Collectively, these findings highlight BNC as a mechanically robust and structurally biomimetic vascular scaffold [18].

### 4.2. Hemocompatibility and Endothelial Response

Hemocompatibility is essential for vascular graft performance, and several authors have demonstrated that BNC supports favorable endothelial behavior. Endothelial cells cultured on BNC shift their transcriptional and proteomic signatures toward quiescent, physiologic phenotypes, reducing inflammatory activation and promoting the expression of markers associated with vascular stability. Implanted BNC-based conduits present minimal fibrin deposition, absence of thrombosis, and progressive endothelialization, while also supporting the development of multilayer vessel-like structures resembling the intima, media, and adventitia. These findings indicate that BNC promotes constructive vascular remodeling rather than fibrosis or occlusion. Furthermore, BNC exhibits low platelet activation, excellent blood cytocompatibility, and high interfacial stability, characteristics that make it particularly suitable for vascular grafts and hemocompatible devices [18,36,50].

### 4.3. Interaction with Vascular and Stromal Cells

BNC supports stromal and vascular cell populations involved in graft maturation. BNC-based scaffolds modified with bioactive molecules such as gelatin have demonstrated improved cellular adhesion and support for stromal and vascular cell interactions, largely through enhanced integrin-mediated attachment and improved biomimetic surface properties. The porous nanofibrillar architecture of BNC further facilitates stromal infiltration and host integration. The porous architecture of BNC allows infiltration of fibroblast-like and smooth muscle–like cells, enabling remodeling of the outer graft layers and progressive integration with host tissue. These phenomena parallel the stromal behavior observed when BNC is used as a cardiac patch, where fibrovascular integration and neovessel formation are consistently reported. The similarity between cardiac and vascular stromal responses suggests that BNC may promote a pro-regenerative, low-inflammation microenvironment across multiple implantation settings [16,18,36].

### 4.4. Mechanical Performance Under Vascular Conditions

Mechanical compatibility between the graft and the host vessel is crucial to prevent anastomotic failure. BNC exhibits non-linear hyperelastic behavior similar to native arteries, supporting its use in vascular environments that undergo cyclic distension. Its tensile strength and resistance to creep allow it to function under continuous pulsatile loading, while composite formulations such as PVA–BNC hydrogels provide anisotropic compliance approaching that of physiological vessels. These properties reduce compliance mismatch and increase long-term graft stability [30].

### 4.5. Hybrid Cardiac–Vascular Constructs

**Figure 3:**
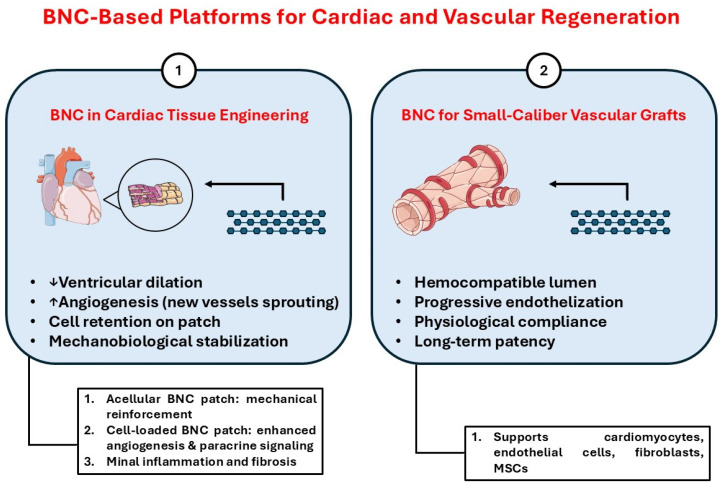
Bacterial nanocellulose (BNC)-based platforms for cardiac and vascular regeneration. BNC supports both myocardial repair and vascular tissue engineering through its adaptable nanofibrillar structure and biocompatibility. In cardiac applications, BNC patches reduce ventricular dilation, promote angiogenesis, enhance cell retention, and provide mechanobiological stabilization, with both acellular and cell-loaded configurations contributing to tissue repair. In vascular applications, BNC enables the fabrication of small-caliber grafts with hemocompatible luminal surfaces, progressive endothelialization, physiological mechanical compliance, and long-term patency. Across both systems, BNC supports multiple cell types, including cardiomyocytes, endothelial cells, fibroblasts, and mesenchymal stem cells, highlighting its role as a unified platform for cardiovascular regeneration.

A major advantage of BNC is its ability to support both cardiomyocytes and endothelial cells, making it highly suitable for the development of prevascularized myocardial constructs. BNC patches implanted on infarcted myocardium induce angiogenesis at the interface between the patch and host tissue, with VEGF-positive neovessels forming within the injured region. Endothelial-compatible BNC surfaces, combined with their mechanical integration with the beating heart, position BNC as a promising substrate for hybrid scaffolds designed to restore both myocardial function and microvascular perfusion [16]. The dual role of BNC in myocardial repair and vascular reconstruction, including its effects on angiogenesis, hemocompatibility, and tissue integration, is summarized in Figure 3.

## 5. Functional Modification of BNC for Cardiovascular Applications

BNC possesses a versatile nanoscale architecture suitable for cardiovascular tissue engineering, but its native biochemical and mechanical characteristics often require refinement to optimize interactions with cardiomyocytes, endothelial cells and stromal populations. Functional modification strategies—including biochemical ligand incorporation, chemical derivatization, structural alignment, and electrical enhancement—allow BNC to emulate key features of cardiac and vascular microenvironments and expand its therapeutic potential.

### 5.1. Biochemical Modification

Biochemical functionalization enhances cell adhesion and signaling by introducing motifs absent in native BNC. Gelatin is one of the most effective bioactive components because it introduces RGD-containing domains that directly engage integrins. Gelatin-mixed BNC (mBNCG) supports markedly higher neonatal cardiomyocyte viability, spreading, and phenotypic maintenance compared with pristine BNC or gelatin-coated membranes, indicating that integrated gelatin improves both biochemical ligand availability and the nanoscale presentation of adhesion sites [34].

Arginine grafting has also been explored as a biochemical strategy. The introduction of positively charged arginine residues onto oxidized BNC enhances electrostatic interactions with ECM proteins and improves the attachment and viability of cardiac and stromal cells. These modifications underscore how biochemical ligand presentation directly shapes cellular adhesion, morphology, and regenerative behavior on BNC substrates [37].

Comprehensive reviews also reinforce that the high functionalization capacity of nanocellulose is a major advantage, enabling the creation of highly tailored microenvironments for cardiac and vascular regeneration [51].

### 5.2. Chemical Modification

Chemical engineering of BNC introduces new functional groups that tune surface charge, hydrophilicity, degradability and protein adsorption. Among these approaches, controlled oxidation is the most established technique. TEMPO-mediated oxidation generates carboxylate (–COO^−^) functionalities that increase negative surface charge and enhance binding of proteins and growth factors [52,53]. This modification improves cardiomyocyte adhesion and promotes compatibility with composite hydrogels. Improved cardiac cell attachment has been observed when oxidation is combined with arginine grafting, illustrating that chemical and biochemical strategies can be synergistic [37].

Additional chemical strategies relevant to biomedical deployment include esterification, etherification, carbamation, and phosphorylation, which modulate mechanical properties and degradability while preserving nanofibrillar integrity. These approaches enable the tailoring of BNC for specific cardiovascular applications, such as myocardial patches requiring prolonged stability or bioresorbable components for transient scaffolding [5].

### 5.3. Structural Modification

Structural refinement of BNC creates microenvironments that guide cell alignment, migration and tissue organization. Alignment of BNC fibers is particularly important for cardiac applications, as cardiomyocytes depend on anisotropic architecture to coordinate force generation and electrical propagation. Electrospun fibrous scaffolds further demonstrate that nanoarchitecture can modulate cardiomyocyte morphology, alignment, and maturation, underscoring the importance of structural design in cardiac regeneration. Studies have demonstrated that aligned cellulose-based matrices promote elongated cardiomyocyte morphology, directional cytoskeletal organization, and improved calcium handling, features consistent with more mature cardiac phenotypes [46,54].

Composite formation is another structural strategy with strong cardiovascular relevance. PVA–BNC hydrogels acquire anisotropic mechanical properties resembling native arterial compliance and nonlinear elasticity, making these materials suitable for vascular and myocardial constructs requiring physiological deformation behavior [27]. Additionally, BNC–starch conduits form functional dual-layer structures with a smooth luminal surface and a porous external region, supporting both blood flow stability and stromal infiltration during graft maturation [50].

Electrospinning of cellulose acetate followed by regeneration into cellulose-based nanofibers represents an additional avenue for structural modification, enabling the creation of fibrous meshes with controlled orientation, nanoscale porosity and mechanical tuning suitable for cardiac cell culture and vascular scaffolding.

### 5.4. Electrical Modification

Cardiac tissue requires synchronized electromechanical conduction; therefore, conductive BNC composites have become a key research direction. Incorporating polypyrrole (PPy) into BNC creates electroactive scaffolds capable of supporting electrical signaling across cultured cardiac cells [5,32,33]. BNC–PPy substrates promote greater cardiomyocyte spreading, enhanced cell–material interaction, and improved differentiation markers compared with non-conductive controls [33]. Conductive enhancement may also be achieved using carbon-based or metallic nanostructures, including graphene, CNTs and gold nanorods. Although not yet broadly tested in vivo for myocardial regeneration, these composite systems show potential to improve electrical propagation within engineered tissues, reduce conduction block and strengthen cell–cell connectivity across regenerative cardiac patches [18].

The use of conductive polymers in cardiac engineering has been widely explored, with evidence showing improved electrical propagation, cell–cell coupling, and cardiomyocyte maturation compared with non-conductive scaffolds [55].

### 5.5. Biological Modification

Biological modification strategies integrate living cells or bioactive components into BNC to create responsive or prevascularized constructs. BNC membranes seeded with skeletal muscle cells and MSCs maintain high viability during culture and, when implanted onto infarcted myocardium, stimulate endothelial proliferation, angiogenesis, and stromal remodeling. These findings suggest that BNC can act as both a structural and biological interface, retaining transplanted cells while facilitating host–graft integration [16].

Emerging work in bacterial engineering further extends this concept. Strategies to genetically program cellulose-producing bacteria enable the synthesis of BNC with embedded peptides or functional biomolecules directly during biosynthesis, providing a route toward intrinsically functional BNC scaffolds that require no post-processing [5].

### 5.6. BNC for Drug Delivery and Biochemical Modulation

The hydrophilicity and nanoscale porosity of BNC enable incorporation and controlled release of therapeutic agents, including anti-inflammatory molecules, antioxidants and pro-angiogenic factors [56,57,58]. Several studies demonstrate that the hydrogen-bonding network of BNC allows sustained release kinetics suitable for cardiac repair, where prolonged local delivery may reduce inflammation, enhance cell survival or stimulate neovascularization [59,60,61]. In myocardial applications, drug-loaded BNC membranes could improve patch performance by modulating fibroblast activity, preventing excessive scarring or supporting long-term engraftment of therapeutic cells [32,33].

These diverse functional modification strategies—biochemical, chemical, structural, electrical and biological—transform BNC into a modular and tunable platform capable of meeting the multifaceted demands of cardiovascular regeneration [60,62,63]. The ability to control adhesion ligand presentation, mechanical anisotropy, electrical conductivity and bioactive molecule retention enables engineered constructs that more closely emulate the myocardial niche and vascular microenvironment. As research progresses, modified BNC systems are positioned to support next-generation myocardial patches, prevascularized cardiac tissues and hybrid bioelectronic implants [60,62,63].

## 6. BNC-Based Bioinks and 3D Bioprinting for Cardiac and Vascular Tissues

The development of bioinks suitable for cardiac and vascular bioprinting requires materials that combine printability, structural fidelity, cytocompatibility, controlled permeability and mechanical adaptability. BNC, due to its nanofibrillar architecture and rheological versatility, has emerged as a promising component for extrusion-based bioinks. Across multiple studies represented in the current literature set, BNC serves primarily as a rheology modifier, mechanical stabilizer, cell-compatible matrix, and structural enhancer within composite hydrogels designed for cardiovascular applications.

Bioprinting has rapidly evolved as a strategy for fabricating personalized tissues, with diverse bioinks, printing geometries, and deposition techniques emerging for cardiac and vascular applications [64,65].

### 6.1. Fundamentals of BNC Bioinks for Cardiovascular Bioprinting

BNC’s nanofibrillar network provides high water-retention capacity, pronounced shear-thinning behavior, and morphological stability, all of which are critical prerequisites for extrusion-based bioprinting. From a rheological perspective, printability depends strongly on the balance between storage modulus (G′) and loss modulus (G″), which governs shape retention after extrusion and flow behavior under shear stress. Ideal cardiovascular bioinks must exhibit sufficient viscoelasticity to maintain filament integrity after deposition while remaining extrudable through clinically relevant nozzle diameters and extrusion pressures without excessive shear-induced cellular damage. Shape fidelity, filament collapse resistance, and post-printing structural stability are therefore central metrics in evaluating BNC-containing bioinks. Crosslinking conditions—including ionic, thermal, or chemical stabilization—also critically influence construct resolution, long-term mechanical integrity, and cell compatibility.

BNC and BNC-derived nanocellulose systems are more commonly incorporated as rheological modifiers or reinforcing components in composite bioinks rather than used as standalone formulations. Nanocellulose–alginate hydrogels exhibit strong shear-thinning behavior, high extrudability, and post-printing stability, with hyaluronic acid further improving shape retention and volumetric fidelity. Printed constructs maintain their architectural features over time, and stem cells (D1-MSCs) preserve viability and proliferation for at least 21 days, confirming the biocompatibility of NC–Alg–HA inks [66]. Gelatin-based hydrogels are widely used in bioink formulations due to their intrinsic RGD motifs and compatibility with cardiac and vascular cell types, supporting the rationale for incorporating BNC into multicomponent printable hydrogel systems [67].

Other studies reinforce this role of BNC as a printability enhancer. Curcumin-loaded cellulose-ester micro-particles incorporated into alginate hydrogels altered viscosity and improved filament formation without compromising cell viability, demonstrating that BNC-derived cellulose systems provide biochemical functionality in addition to structural reinforcement [68]. Similarly, broader bioink reviews and nanocellulose-based bioprinting studies indicate that nanocellulose-containing formulations can improve extrusion consistency, modulate flow behavior, and support multilayer deposition through rheological reinforcement [64,65,69].

Taken together, these findings establish BNC as a key rheological component enabling the fabrication of cardiac and vascular constructs with high definition and biological performance.

### 6.2. Rheology, Printability and Structural Fidelity of BNC Bioinks

The rheological performance of BNC-containing bioinks is dominated by two core features: shear-thinning behavior and nanofibril-mediated entanglement. Shear-thinning is essential for cardiac bioprinting because it enables low-viscosity extrusion under pressure while maintaining high viscosity post-printing to preserve construct geometry.

To address printability more critically, BNC-containing bioinks should be evaluated using quantitative rheological and processing parameters rather than only qualitative descriptors. These include storage modulus (G′), loss modulus (G″), G′/G″ ratio, shear-thinning behavior, extrusion pressure, nozzle diameter, crosslinking method, shape fidelity, and post-printing cell viability [64,65].

The addition of BNC significantly increases the storage modulus (G′) and enhances structural recovery after shear, resulting in 3D constructs with superior fidelity and resistance to collapse. Hyaluronic acid further improves these properties by increasing water retention and hydrogel consistency. The resulting NC–Alg–HA scaffolds maintain their strut height, width, and pore geometry across multiple layers, a requirement for complex cardiovascular tissues that depend on hierarchical internal architecture. Sterilization is a major constraint in bioink preparation, as autoclaving frequently disrupts hydrogel microstructure; however, BNC composite bioinks exhibit notable stability under short-cycle autoclave conditions. These systems preserve viscosity and printability after sterilization, enabling the safe preparation of cell-laden formulations [66].

Studies on cellulose-derived hydrogels, including those using cellulose acetate nanofibers, reveal that structural alignment and controlled crosslinking (particularly Ca^2+^ crosslinking of alginate) generate scaffolds with anisotropy suitable for cardiac constructs and mechanical compliance relevant to vascular tissues [70].

Drug-loaded cellulose ester hydrogels add another dimension: BNC-related materials can encapsulate bioactive molecules without disrupting rheology, creating multifunctional inks capable of delivering anti-inflammatory or antioxidant agents during tissue maturation. Collectively, rheological and structural evidence positions BNC as a robust stabilizer of bioinks for high-resolution, multi-layered cardiac and vascular constructs [68].

### 6.3. BNC-Enhanced Bioinks for Vascular Bioprinting

Vascular bioprinting requires materials that support luminal smoothness, endothelial adhesion, and radial mechanical stability. Composite BNC bioinks address these requirements by improving filament rigidity and reducing sagging, thereby enabling the printing of tubular conduits with controlled wall thickness. BNC-based vascular grafts, including starch-reinforced BNC conduits, exhibit a bilayer architecture with a smooth luminal surface and a porous outer layer that mimics native arterial structure and supports endothelialization. When translated into bioprinting, these characteristics are reflected in enhanced viscosity and scaffold stability, facilitating the fabrication of structurally reliable vascular constructs [50].

Nanocellulose-supported alginate and hyaluronic acid inks promote endothelial viability and alignment, which are critical for the formation of functional vascular linings. NC–Alg–HA constructs support sustained MSC viability and extracellular matrix deposition, serving as essential precursors for vessel wall maturation [66].

Recent advances include bioinks embedding drug-loaded cellulose ester particles, enabling the fabrication of vascular grafts with intrinsic anti-inflammatory or antioxidant properties [68]. Such ink systems could be particularly useful in small-diameter vascular conduits where inflammatory restenosis is a major limitation. Together, these results show that BNC-based bioinks meet the mechanical, structural and biological requirements needed for printing vascular tissues capable of endothelialization, perfusion, and long-term stability.

### 6.4. BNC-Enhanced Bioinks for Myocardial and Cardiac Tissue Bioprinting

Cardiac tissue bioprinting demands scaffolds with anisotropic architecture, electromechanical support, and compatibility with cardiomyocytes. BNC-based bioinks contribute to these goals in several ways. First, the nanofibrillar structure of BNC promotes fiber alignment, which can be leveraged by directional printing to induce cardiomyocyte alignment. Cellulose-based aligned fiber scaffolds have been shown to support elongated cardiomyocyte morphology and improved calcium cycling [46]. When incorporated into printed hydrogels, BNC nanofibrils serve as templates for reproducible anisotropy.

Second, composite BNC bioinks can accommodate conductive materials. BNC–polypyrrole composites enhance cardiomyoblast adhesion and promote cytoskeletal extension compared with non-conductive BNC, suggesting that conductive bioinks incorporating BNC may support synchronous contraction in printed cardiac tissues [45].

Third, BNC improves the mechanical integrity of cardiac patches printed using alginate- or HA-based matrices. Cardiomyocytes, MSCs, and endothelial cells survive well within NC-enhanced hydrogels and exhibit morphological characteristics consistent with early tissue organization. Sustained MSC viability over 21 days within NC–Alg–HA constructs supports the capacity of these systems to maintain cell growth and differentiation in cardiac environments [66].

Fourth, BNC-derived components enable bioactive ink design, as demonstrated by the Curcumin-loaded bacterial cellulose–gelatin composite hydrogels, which further demonstrate that BNC-derived systems can incorporate bioactive molecules while maintaining favorable biological compatibility, supporting their potential as multifunctional biofabrication platforms [68].

Finally, because BNC is compatible with autoclave sterilization and does not collapse under thermal processing, it is suitable for producing sterile, cell-ready cardiac bioinks, a major limitation for protein-based inks such as collagen or Matrigel.

Overall, current evidence supports BNC bioinks as promising candidates for developing anisotropic, conductive and biologically stable myocardial constructs, capable of supporting cardiomyocyte alignment, stromal integration and eventual electromechanical coupling.

## 7. Challenges, Limitations, and Future Perspectives

Although BNC has demonstrated compelling performance in cardiovascular applications, several scientific, technological and translational barriers still limit its full transition to clinical practice. Understanding these challenges is essential for guiding advances in scaffold design, biological integration, manufacturability and regulatory readiness. Additional translational challenges extend beyond biological performance. Large-scale clinical translation of BNC-based cardiovascular biomaterials will require GMP-compliant manufacturing pipelines capable of ensuring batch-to-batch reproducibility in fibril architecture, hydration behavior, mechanical properties, and functionalization consistency. Endotoxin contamination remains a critical concern given the bacterial origin of BNC, requiring rigorous purification and quality-control protocols. Sterilization strategies also present important trade-offs, as thermal, irradiation, or chemical sterilization may alter nanofibrillar structure, mechanical compliance, or bioactivity. Standardized manufacturing and regulatory benchmarking will therefore be essential before widespread cardiovascular translation becomes feasible.

### 7.1. Biological and Biochemical Limitations

One fundamental limitation of native BNC is its lack of intrinsic biochemical ligands required for optimal cardiomyocyte adhesion and maturation. Although protein adsorption can partially support cell attachment, robust cardiac applications generally require biochemical functionalization, such as gelatin incorporation, peptide grafting, oxidation, or arginine-based modification. These strategies improve bioactivity but also increase manufacturing complexity, batch-to-batch variability, and regulatory burden [34,37].

Another challenge is the non-degradability of BNC in mammalian tissues. While long-term stability is beneficial for mechanical reinforcement, such as infarct stabilization or vascular conduits, it may hinder complete tissue remodeling in applications requiring gradual scaffold resorption. Strategies to introduce controlled degradability through oxidation, enzymatically cleavable linkers or hybrid formulations remain under development and require careful tuning to avoid compromising mechanical integrity [5].

These challenges are consistent with broader discussions regarding the barriers that emerging biomaterials such as nanocellulose still face before achieving large-scale clinical translation [71].

### 7.2. Mechanical and Structural Challenges

Although BNC exhibits biomimetic mechanical properties, achieving precise anisotropy and viscoelastic tuning remains essential for advanced myocardial constructs. Cardiomyocytes rely on aligned fibrillar scaffolds to promote directional contraction and physiological electromechanical coupling. While aligned cellulose constructs have shown promise in supporting cytoskeletal organization and calcium dynamics, the large-scale fabrication of anisotropic BNC structures with consistent mechanical gradients remains technically challenging [46].

Similarly, vascular grafts must match the compliance of native vessels to prevent anastomotic hyperplasia. While PVA–BNC composites approximate arterial elasticity and BNC–starch conduits maintain patency under physiological pressures, further refinement is required to ensure long-term fatigue resistance and mechanical stability under pulsatile flow [27,50,72].

### 7.3. Electromechanical Integration

BNC is inherently electrically inert, which limits its ability to support synchronous excitation–contraction coupling in engineered cardiac tissues. Conductive modifications, such as polypyrrole integration [45] or the incorporation of conductive nanomaterials, may enhance electrical propagation; however, their long-term biocompatibility, degradation profile, and electrical stability in vivo remain insufficiently characterized. For clinical translation, conductive BNC scaffolds must demonstrate safe conduction, absence of arrhythmogenic risk, and stable integration with host myocardium.

### 7.4. Vascularization and Tissue Integration

Thick cardiac constructs require rapid and sustained vascularization to ensure nutrient delivery and cell survival. Although BNC patches promote angiogenesis in infarct models, achieving prevascularized or perfusable networks within engineered tissues remains an open challenge. Strategies incorporating endothelial cells, angiogenic factors, or BNC-based bioinks capable of printing patterned microchannels offer promising directions but require validation in large-animal models and under physiological loading [16,66].

### 7.5. Manufacturing, Sterilization and Scalability

The microbial production of BNC is a major advantage, but GMP-standard manufacturing remains a key challenge. Variability in fermentation conditions, purification steps, and post-processing can affect nanofibril density, crystallinity, and mechanical performance. In addition, while short-cycle autoclaving preserves the properties of BNC-based bioinks, consistent sterilization protocols for BNC membranes, grafts, and composite scaffolds require further standardization. Additives such as gelatin, growth factors, or conductive polymers introduce additional complexity, emphasizing the need for regulatory-compliant purification and rigorous verification of bioburden, endotoxin levels, and residual reagents [66].

### 7.6. Regulatory and Translational Barriers

The translation of BNC cardiovascular implants faces significant regulatory hurdles. Devices such as cardiac patches, vascular conduits or bioink-derived tissues must demonstrate: long-term biocompatibility; hemocompatibility and endothelial stability; controlled mechanical performance under cyclic loading; absence of cytotoxic or proarrhythmic effects, sterility and manufacturing reproducibility, and efficacy in large-animal models, not only rodents [34,73,74,75].

To date, BNC has been clinically approved primarily for skin regeneration applications, which involve far lower mechanical and hemodynamic demands. Vascular and myocardial applications will require rigorous preclinical validation and comparative performance testing against established biomaterials such as ePTFE, Dacron and biological grafts [5].

### 7.7. Future Perspectives

Despite these challenges, BNC remains one of the most versatile and promising biomaterials for cardiovascular engineering. Near-term translational opportunities include acellular BNC cardiac patches for mechanical stabilization after myocardial infarction [16,76]; cell-loaded or growth factor–modified BNC membranes to enhance regenerative signaling [77,78]; small-caliber BNC-based vascular grafts that leverage their patency and hemocompatibility [50,79]; BNC-enhanced bioinks for anisotropic myocardial constructs and vascularized tissues [66]; conductive BNC composites to improve excitation propagation in engineered myocardium [79,80,81]; and hybrid BNC systems for drug delivery enabling sustained therapeutic release in ischemic environments.

Collectively, advances in biochemical functionalization, conductive modification, composite mechanics and 3D bioprinting are rapidly establishing BNC as a next-generation platform for myocardial regeneration and vascular tissue construction. Continued progress in mechanobiology, biomanufacturing and regulatory sciences will be decisive for unlocking its clinical translation potential. Another promising future direction is the development of hybrid BNC–cardiac dECM hydrogels. In this strategy, BNC provides mechanical reinforcement, hydration, and fibrillar stability, whereas cardiac dECM contributes tissue-specific biochemical signals such as collagen, fibronectin, laminin, and elastin. Such composites may help bridge the gap between mechanically robust BNC scaffolds and biologically instructive myocardial matrices, particularly for cardiac patches, injectable hydrogels, and bioink platforms.

## Figures and Tables

**Table 1:** Comparative physicochemical, mechanical, and biological features of representative BNC-based cardiovascular systems.

BNC-Based System	Main Application	Key Mechanical/Physicochemical Feature	Biological/Functional Relevance
Native BNC	Myocardial patch/structural scaffold	High crystallinity, hydrated nanofibrillar network, high stiffness and fatigue resistance	Mechanical stabilization of infarcted myocardium; support for tissue integration
Gelatin-mixed BNC	Cardiomyocyte culture scaffold	Elastic modulus approximately 75 kPa; improved nanoscale roughness	Enhanced cardiomyocyte adhesion, spreading, and morphology
PVA–BNC composite	Vascular/arterial-like construct	Anisotropic compliance and nonlinear strain response	Better approximation of arterial mechanics
BNC/starch conduit	Small-caliber vascular graft	Bilayer structure; smooth luminal surface; microporous outer layer	Approximately 75% in vivo patency; resistance to thrombogenicity and aneurysmal deformation
NC–Alg–HA bioink	3D bioprinting	Shear-thinning behavior, high extrudability, post-printing shape stability	Maintains printed architecture; supports MSC viability/proliferation for at least 21 days
BNC–PPy conductive composite	Cardiac electroactive scaffold	Conductive modification with polypyrrole	Improved cardiomyoblast spreading, filopodia formation, and cardiac-like phenotype

**Table 2:** Preclinical Applications of Bacterial Nanocellulose (BNC) Systems in Cardiac Regeneration.

System/Configuration	Cell Type(s)	Main Findings Relevant to Regeneration
BNC patch (acellular)	–	Preserves LV dimensions, limits post-infarction remodeling, integrates with minimal inflammation [16].
BNC patch + cocultured cells	Skeletal muscle cells + MSCs	High in vitro viability, engraftment on patch, increased proliferation and angiogenesis in infarct zone [16].
Gelatin-coated BNC (BCG)	Neonatal rat cardiomyocytes	Improved adhesion and viability versus pure BNC; partial mechanical softening [34].
Gelatin-mixed BNC (mBCG)	Neonatal rat cardiomyocytes	Highest viability, appropriate stiffness and roughness, best support for cardiomyocyte spreading and morphology [34].
BNC–PPy conductive composite	H9c2 cardiomyoblasts	May support normal cell morphology, increases filopodia and cell density, enhances differentiation toward cardiac phenotype [45].

## Data Availability

No new data were created or analyzed in this study. Data sharing is not applicable to this article.
